# Seasonal Plasticity in Tryptophan Metabolism Provides New Insights Into Physiological Adaptation in Snake Hibernation

**DOI:** 10.1002/ece3.72202

**Published:** 2025-09-17

**Authors:** Yuting Wei, Zexiu Zhang, Xiaohong Lin, Gangning Wei, Pengyue Zhang, Huirong Mao, Biao Chen, Jianhao Ji, Yunlin Zheng, Zhiyi Luo, Xiaolong Hu

**Affiliations:** ^1^ College of Animal Science and Technology Jiangxi Agricultural University Nanchang China; ^2^ Jiangxi Provincial Key Laboratory of Conservation Biology Jiangxi Agricultural University Nanchang China; ^3^ Zhangzhou Pien Tze Huang Pharmaceutical Co., Ltd Zhangzhou China

**Keywords:** hibernation, indoles, kynurenic acid, snake, tryptophan

## Abstract

Hibernation is a common behavioral strategy for snakes to cope with extreme environments. This phenomenon has raised important scientific questions regarding its physiological adaptation mechanisms. Although tryptophan and its metabolites have been widel y studied for their roles in various physiological processes in animals—including immune regulation, metabolic homeostasis, and circadian rhythms—the impact of snake hibernation on tryptophan metabolism remains unexplored. In the present study, an integrated multi‐omics approach that combines targeted metabolomics, transcriptomics, and microbiome was used to reveal the tryptophan metabolism mechanisms in active and hibernating snakes. Our results revealed that the higher gut indole concentrations observed in active snakes indicate a greater reliance on microbial pathways in their tryptophan metabolism. Correlation analyses between gut microbiota and indole levels further identified specific bacterial genera—*Paeniclostridium, Romboutsia*, and *Clostridium sensu stricto 1*—as potential key contributors to tryptophan conversion into indole. Additionally, the higher serum concentrations of metabolites such as kynurenic acid and 5‐hydroxytryptophol, along with the upregulated expression of key genes, indicate that hibernating snakes exhibit an increased reliance on the kynurenine and 5‐hydroxytryptamine pathways for tryptophan metabolism. These findings collectively suggested that the seasonal plasticity of tryptophan metabolism may mediate physiological adaptations during snake hibernation, thereby providing deeper cognition into the mechanisms underlying reptilian hibernation strategies.

## Graphical abstract

The microbial pathways of tryptophan metabolism predominate in active snakes, while the 5‐hydroxytryptamine pathway and kynurenine pathway govern tryptophan catabolism in hibernating snakes.

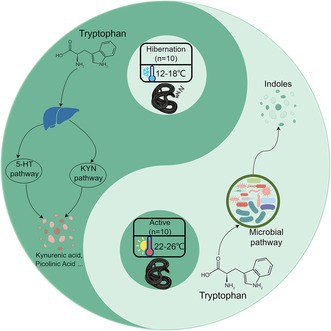

## 1 Introduction

Hibernation is a remarkable physiological adaptation observed in snakes, various other ectotherms, and some mammals (Staples 2016). It involves a significant reduction in metabolic rate and body temperature to survive the harsh conditions of winter (Geiser 2013; Secor and Carey 2016). During this period of torpor, hibernators face unique physiological challenges, including potential immunosuppression and oxidative stress due to altered metabolism and prolonged inactivity. The initiation and maintenance of hibernation in animals are closely regulated by the circadian clock system, which orchestrates metabolic and behavioral adaptations through transcriptional‐translational feedback loops (Gavrić et al. 2017; Lutterschmidt 2012; Spence et al. 2020). Recent research has underscored the unique roles of tryptophan metabolites in regulating circadian rhythms, enhancing immune responses, exerting antioxidant activity, and maintaining metabolic homeostasis (Feng et al. 2022; Zhang et al. 2022). The complex interplay between the tryptophan metabolism and the hibernation process indicates the resilience of these animals in extreme environmental conditions. Additionally, it offers valuable knowledge into the physiological mechanisms that help vertebrates manage various stresses.

Tryptophan is an essential aromatic amino acid for humans and animals, and it serves as a precursor for various bioactive compounds. Its metabolic pathways include the serotonin (5‐hydroxytryptamine) synthesis pathway, kynurenine (KYN) pathway, and microbial catabolism (indoles pathway) (Xue et al. 2023). In the nervous system, tryptophan is hydroxylated by tryptophan hydroxylase to produce serotonin, which is further converted into melatonin. These two metabolites play crucial roles in regulating mood and modulating circadian rhythms, particularly in managing sleep–wake cycles (Versteeg et al. 2015). Beyond neuromodulation, tryptophan metabolism is closely linked to immune responses and energy homeostasis. KYN pathway metabolites, such as quinolinic acid and kynurenic acid (KA), exhibit immunomodulatory properties by influencing T‐cell differentiation and inflammatory reactions (Martin et al. 2020; Seo and Kwon 2023). At the protein synthesis level, tryptophan is involved in the mTOR signaling pathway to regulate cellular growth and repair, thereby promoting muscle development and injury recovery (Tang et al. 2020). Recent studies reveal a bidirectional regulatory relationship between tryptophan metabolism and gut microbiota (Agus et al. 2018). Specific microbial communities catabolize tryptophan to generate indole derivatives, which modulate host immunity and metabolic homeostasis (Dong et al. 2020). These findings underscore the central role of tryptophan metabolism in physiological regulatory networks, providing a theoretical foundation for investigating its functions in specialized physiological states, such as hibernation.

Although tryptophan metabolism has been extensively studied in endotherms, its role in hibernation among ectotherms remains unexplored. During snake hibernation, the extreme suppression of energy metabolism and a significant reduction in neural activity may correlate with adaptive adjustments in tryptophan metabolic pathways. To rigorously validate the above hypothesis, this study will utilize an integrated multi‐omics approach that combines serum‐targeted metabolomics, hepatic transcriptomics, gut microbiota analysis, and targeted metabolomics. This comprehensive strategy aims to explore the variations in tryptophan metabolism between hibernation and active periods in snakes, examining changes in gene expression, metabolic products, and microecological dynamics. Finally, the study aims to elucidate mechanisms by which snakes leverage tryptophan metabolism to adapt to hibernation.

## 2 Materials and Methods

### 2.1 Animal Grouping and Sample Collection

The Jiangxi Agricultural University approved the animal experiments (approved number: JXAU#2024D021), which were conducted in compliance with the existing regulations on animal welfare and research in China.

Experiments were conducted from January to February 2025 using 20 male Chinese Moccasins ( *Deinagkistrodon acutus* ) with similar body weights (Table S1). The snakes were evenly divided into two groups. Chinese Moccasins typically hibernate from November to April of the following year. During hibernation, individuals seek sheltered environments to maintain temperatures above 10°C, thereby avoiding freezing‐related mortality. From May to October, the snakes are in an active state, with an optimal temperature range of 22°C–28°C. Accordingly, the present study established two temperature conditions to represent the active and hibernation periods: the Active group was housed in box‐type enclosures equipped with heating devices maintaining temperatures between 22°C and 26°C, while the Hibernation group was kept in enclosures without heating where temperatures fluctuated with ambient conditions between 12°C and 18°C. The snakes were routinely fed ground cull chicks purchased from a poultry farm, with active snakes fed once every 7 days. Hibernating snakes were not fed, as they naturally cease voluntary feeding under ambient temperatures below 20°C. All active snakes were fasted for 1 week prior to sampling. Temperature parameters were continuously monitored and recorded throughout the experiment using a fully automated temperature, humidity, and light monitoring system (Elitech GSP‐8G). According to the temperature monitoring records, the hibernating snakes had been maintained within the range of 12°C–18°C for one and a half months prior to sampling.

Sample collection for physiological analysis was conducted on 25 February. Initially, the blood sample was collected from the snake cloaca using a syringe. The blood was then transferred to centrifuge tubes without anticoagulant, and 20 serum samples (about 4 mL each sample, *n* = 10) were collected following the centrifugation of blood. Subsequently, the snakes were humanely euthanized via decapitation, and 20 liver samples (about 2 g each sample, *n* = 10) were collected from each snake. Finally, luminal contents from the entire intestinal segments (at least 1 g) were collected by extruding the contents into tubes using a sterile spatula. A total of 40 luminal samples were collected in duplicate for microbial 16S rRNA gene sequencing (*n* = 10) and metabolome (*n* = 10), respectively. All samples were temporarily stored in liquid nitrogen and finally stored in a −80°C refrigerator after transportation to the laboratory.

### 2.2 Gut 16S rRNA Gene Sequencing

DNA was purified using a commercial kit (QIAamp DNA Stool Mini Kit, Qiagen) following the manufacturer's instructions. This process removed contaminants and ensured a high yield and quality of DNA. The V3‐V4 region of the 16S rRNA gene was amplified using universal primers (341F and 805R) that are compatible with Illumina sequencing platforms. The amplified PCR products were quantified with a fluorometric assay (Qubit, Thermo Fisher) to maintain a consistent library concentration. The pooled library was sequenced on an Illumina MiSeq platform using a 2 × 300 bp paired‐end sequencing strategy to generate high‐quality sequence reads.

Raw sequence reads were quality‐filtered using Quantitative Insights into Microbial Ecology 2 (QIIME2, Bolyen et al. 2019). This process involved removing reads with average quality scores below a threshold and truncating reads at the first occurrence of a base with a quality score below that threshold. The quality‐filtered reads were clustered into amplicon sequence variants (ASVs) using the Divisive Amplicon Denoising Algorithm 2 (DADA2) in the QIIME 2 pipeline (Callahan et al. 2016). Taxonomic classifications for ASVs were assigned using the SILVA 138 database. Alpha and beta diversity metrics were calculated to assess community richness, diversity, and similarity in QIIME2. Principal coordinate analysis (PCoA) based on Bray–Curtis dissimilarity was conducted alongside nonparametric multivariate analysis of variance (ADONIS), while nonmetric multidimensional scaling (NMDS) based on weighted UniFrac distance was performed in combination with analysis of similarity (ANOSIM) via R (www.rproject.org). Additionally, Linear discriminant analysis effect size (LEfSe) was performed in QIIME2. Phylogenetic Investigation of Communities by Reconstruction of Unobserved States 2 (PICRUSt2) software was used for function annotation analysis (Douglas et al. 2020). StampPlots were generated to visualize effect sizes of these functions, with coefficients deemed significantly affected when their 95% confidence intervals excluded zero—indicating either a positive (all values > 0) or negative (all values < 0) effect. The *t*‐test (for normal data) or Mann–Whitney *U* test (for discrete data) was used to calculate the significance of microbial α‐diversity and dominant microbial taxa in Statistical Package for the Social Sciences software (SPSS version 23.0; IBM, Chicago, IL, USA).

### 2.3 Targeted Metabolome of Gut Indoles and Serum Tryptophan Metabolites

Frozen gut content was mixed with 10 pre‐chilled zirconium oxide beads and 20 μL of deionized water. The above mixture and serum samples were homogenized in a mixture of methanol and water (80:20, v/v), respectively. This mixture choice was effective for extracting a wide range of metabolites, including indoles. After homogenization, samples were centrifuged at 10,000 rpm for 10 min at 4°C. The supernatant was collected and filtered through 0.22 μm syringe filters to remove particulate matter. An internal standard solution was added to each sample to monitor and correct for variations in extraction efficiency and instrument response. The extracted samples were analyzed using a High‐Performance Liquid Chromatography system coupled with a triple quadrupole tandem mass spectrometry detector for the targeted quantification of indoles and tryptophan metabolites. Calibration curves were generated using a series of standards to establish a linear relationship between the analyte concentration and the peak area. Quality control samples were analyzed alongside the unknown samples to ensure the accuracy and precision of the method.

Raw data were processed using specialized software to integrate chromatographic peaks and quantify metabolites based on calibration curves. Metabolite concentrations were normalized to the internal standard to account for variations in sample preparation and instrument performance. Normality of each variable was assessed using the Shapiro–Wilk test, with a threshold of *p* > 0.05 indicating a normal distribution. Data following a normal distribution were analyzed using independent‐samples t‐tests, while non‐normally distributed data were assessed using the Mann–Whitney *U* test, and the statistical significance was defined as *p* < 0.05. The specific statistical method applied to each parameter is indicated in the results section. The data visualization was presented as box plots in the OmicStudio platform (Lyu et al. 2023).

### 2.4 Liver Transcriptome

Frozen liver tissues were thawed on ice and homogenized in TRIzol reagent using a tissue homogenizer to ensure effective cell lysis and RNA release. Total RNA was isolated following the manufacturer's instructions for the TRIzol method. RNA was further purified using an RNA cleanup kit (RNeasy Mini Kit, Qiagen) to remove genomic DNA and other contaminants. RNA integrity was assessed with an Agilent Bioanalyzer, and RNA concentration was measured using a Nanodrop spectrophotometer or a Qubit fluorometer. Adapter‐ligated cDNA libraries were amplified using PCR to generate sufficient quantities for sequencing. PCR conditions were optimized to avoid over‐amplification and bias. Finally, the libraries were sequenced on a high‐throughput sequencing platform (Illumina HiSeq) to produce paired‐end reads of a specified length.

Raw sequencing reads were filtered using Trimmomatic (Bolger et al. 2014) to eliminate adapter sequences, low‐quality bases, and ambiguous bases. Clean reads were mapped against the reference genome, and the transcript abundances were quantified using Kallisto (Bray et al. 2016). Differential expression analysis was performed using DESeq2 to identify genes that were significantly upregulated or downregulated (Love et al. 2014). The differentially expressed genes were annotated using databases such as gene ontology (GO) and Kyoto encyclopedia of genes and genomes (KEGG), which helped determine their biological functions and pathways. After screening for genes related to tryptophan metabolism, the upregulated and downregulated differential genes were identified by Volcano plots based on log2(FC) and −log10(*p* value) in the OmicStudio platform (Lyu et al. 2023).

### 2.5 Correlation Analysis

To investigate the relationship between gut microbiota and gut indole metabolites, as well as serum tryptophan metabolites, the correlation network heatmap analysis was performed. This analysis utilized the relative abundance table of gut microbiota and the concentration table of differentially expressed serum and gut metabolites on the OmicStudio platform (Lyu et al. 2023). Subsequently, the correlation between differentially expressed liver genes and serum metabolites was performed to identify the specific genes involved in the certain differential tryptophan metabolism pathways in R. Finally, to clearly delineate which tryptophan metabolic pathway predominates during the active or hibernation periods, the hand‐painted heatmap pathway maps were produced by Figdraw (www.figdraw.com). These maps were based on the serum tryptophan metabolite data, gut indole profiles, and hepatic transcriptomic datasets obtained from this study, focusing on the three established tryptophan metabolic pathways.

## 3 Results

### 3.1 Analysis of Gut Microbiota

**FIGURE 1:**
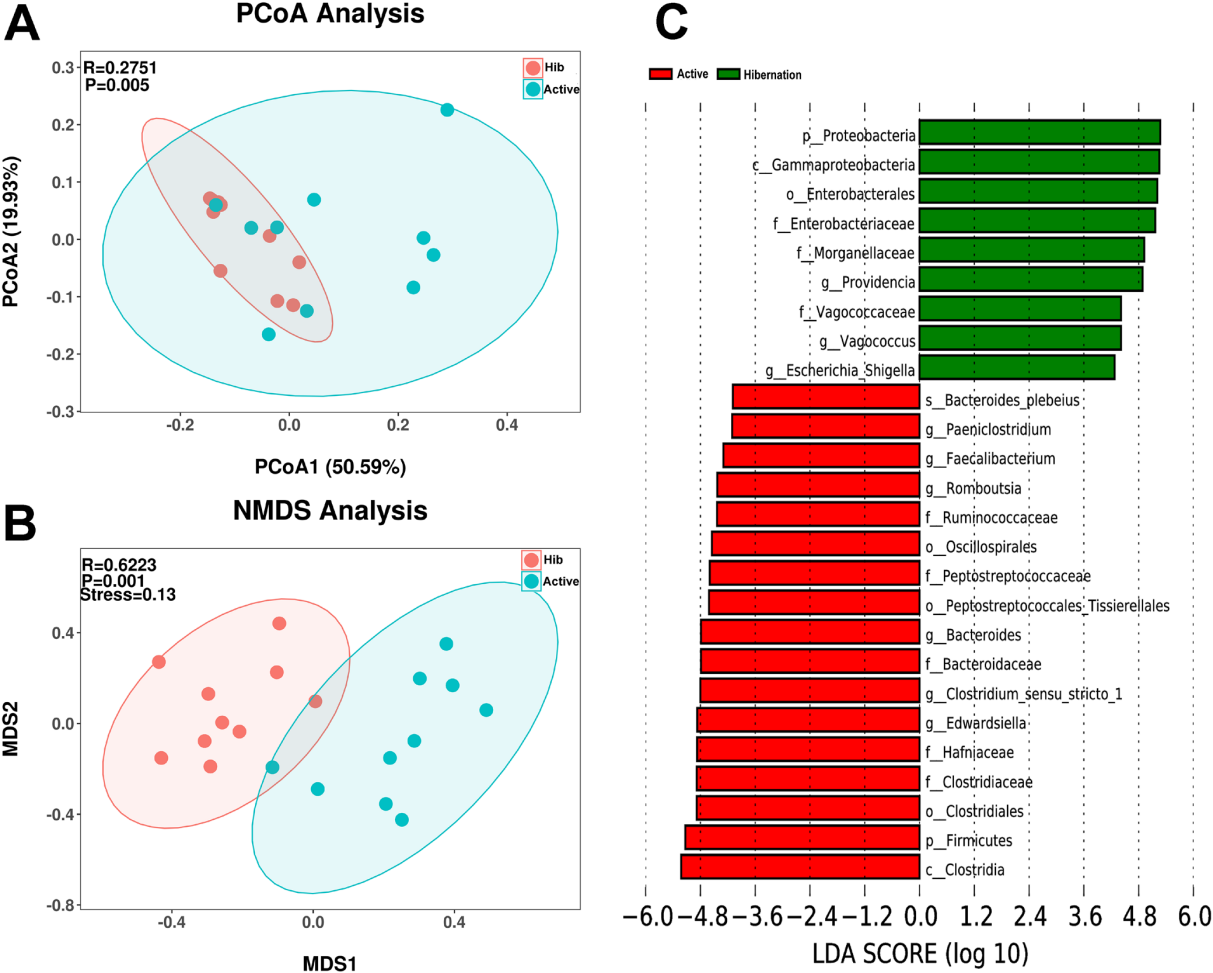
The comparison of gut microbial community based on the β‐diversity in Chinese Moccasins. Principal‐coordinate analysis (PCoA, A) and the nonmetric multidimensional scaling (NMDS, B) are generated to show the different distributions of samples. Red colors in plots refer to samples of hibernating snakes (Hib), and blue colors refer to samples of active snakes (Active). *R* values and *p* values in PCoA and NMDS are calculated by nonparametric multivariate analysis of variance (ADONIS) and analysis of similarity (ANOSIM), respectively. The linear discriminant analysis (LDA) plot (C) shows the different taxa at six levels upon the threshold of 4. Letters before the taxa names are the phylum (p), class (c), order (o), family (f), genus (g), and species (s).

All indices of microbial α‐diversity exhibited non‐significant differences between hibernation and active snakes (Table S2, Figure S1). Although PCoA results based on weighted Unifrac distance indicated an overlap between the active and hibernating snakes (Figure 1A), the ANOSIM revealed significant differences. Furthermore, the NMDS plot displayed distinct clusters between the two groups (Figure 1B), while the ANOSIM confirmed the significant differences. The LEfSe analysis, using a threshold of 4, identified 26 microbial taxa with significant differences between the two groups (Figure 1C). This included 2 phyla, 2 classes, 4 orders, 8 families, 9 genera, and 1 species. Meanwhile, the core microbe analysis identified different core microbes between the two groups, particularly in the phyla Proteobacteria and Firmicutes (Figure S2).

**FIGURE 2:**
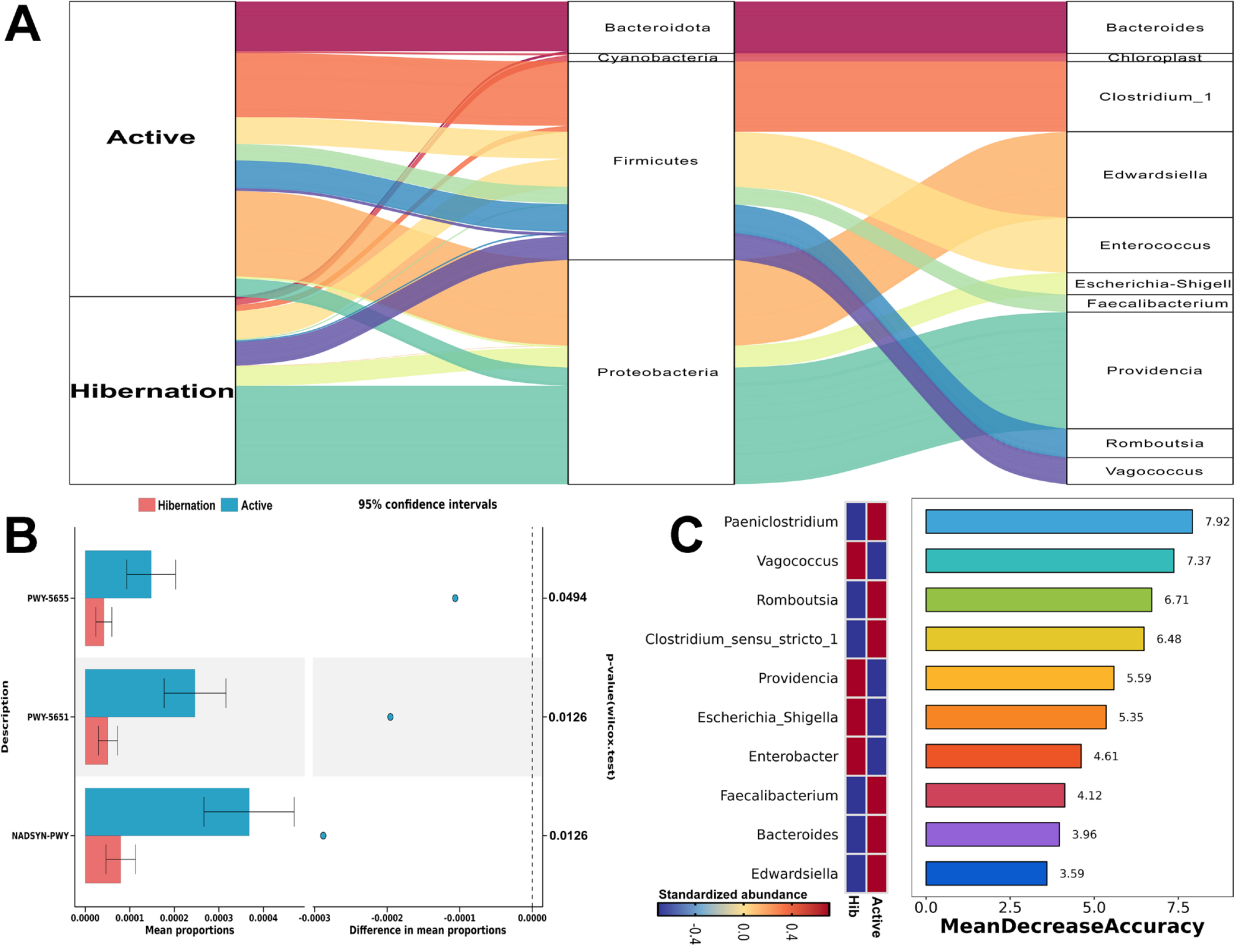
The comparison of gut microbial composition and function in Chinese Moccasins. The Sankey plot (A) shows the dominant microbial phyla and genera in different sampling groups and the relationship between phyla and genera. Comparison (B) of the three significantly different microbial functions related to tryptophan metabolism identified by Phylogenetic Investigation of Communities by Reconstruction of Unobserved States 2 (PICRUSt2). Random forest analysis (C) of gut bacterial community. The *y*‐axis, from top to bottom, displays the genera ranked by their relative importance based on mean decrease accuracy in the classification of groups. Hib, samples from hibernating snakes; Active, samples from active snakes.

Taxonomic analysis indicated that the Proteobacteria, Firmicutes, and Bacteroidota were the dominant microbial phyla in all the 20 snakes (the middle column in Figure 2A). The dominant genera are detailed in the right column of Figure 2A. The quantitative comparison of dominant phyla demonstrated that the relative abundance of Proteobacteria was higher in hibernation snakes, while Firmicutes was more prevalent in active snakes (Table 1). Among the dominant genera, *Providencia*, *Vagococcus*, and *Escherichia_Shigella* demonstrated higher abundance in hibernation snakes, while *Edwardsiella*, *Romboutsia*, *Bacteroides*, *Clostridium_sensu_stricto_1*, *Faecalibacterium*, and *Paeniclostridium* displayed higher abundance in active snakes (Table 1). Among the dominant species, *Bacteroides_fragilis* and *Roseburia_inulinivorans* depicted higher abundance in active snakes (Table 1).

**TABLE 1:** The relative abundance (%, mean ± SE) and statistics of dominant microbial phyla, genera and species among active and hibernation snakes. The *p* values were calculated by independent‐samples *t*‐test or nonparametric Mann–Whitney *U*‐test.

Taxa	Group		Significance
	Active	Hibernation	
*Phylum*			
Proteobacteria	34.01 ± 9.68	70.17 ± 6.50	*t* = 3.10, *p* = 0.006
Firmicutes	50.81 ± 7.99	23.44 ± 6.52	*t* = 2.65, *p* = 0.016
Bacteroidota	13.21 ± 4.98	1.88 ± 0.62	*U* = 37.00, *p* = 0.353
*Genus*			
*Providencia*	4.14 ± 3.48	22.38 ± 6.21	*U* = 15.00, *p* = 0.007
*Edwardsiella*	19.47 ± 9.73	0.05 ± 0.03	*U* = 23.00, *p* = 0.043
*Romboutsia*	6.26 ± 2.99	0.31 ± 0.10	*U* = 5.00, *p* < 0.001
*Bacteroides*	11.28 ± 4.65	0.54 ± 0.35	*U* = 14.00, *p* = 0.005
*Vagococcus*	0.63 ± 0.17	5.43 ± 1.31	*U* = 6.00, *p* < 0.001
*Clostridium_sensu_stricto_1*	14.58 ± 6.95	1.35 ± 0.70	*U* = 7.00, *p* < 0.001
*Enterococcus*	6.11 ± 3.54	6.44 ± 3.36	*U* = 47.00, *p* = 0.853
*Faecalibacterium*	3.73 ± 1.21	0.23 ± 0.12	*U* = 17.00, *p* = 0.011
*Paeniclostridium*	3.09 ± 1.01	0.10 ± 0.05	*U* = 4.00, *p* < 0.001
*Escherichia_Shigella*	0.47 ± 0.12	4.51 ± 1.98	*U* = 12.00, *p* = 0.003
*Species*			
*Clostridium_moniliforme*	8.89 ± 6.27	0.57 ± 0.52	*U* = 32.50, *p* = 0.190
*Bacteroides_fragilis*	0.86 ± 0.42	0.12 ± 0.09	*U* = 22.00, *p* = 0.035
*Bacteroides_plebeius*	2.21 ± 1.66	0.01 ± 0.004	*U* = 25.00, *p* = 0.063
*Clostridium_sp*	0.82 ± 0.47	0.04 ± 0.01	*U* = 31.00, *p* = 0.165
*Bacteroides_coprocola*	0.84 ± 0.50	0.01 ± 0.01	*U* = 27.00, *p* = 0.089
*Bacteroides_stercoris*	0.78 ± 0.42	0.01 ± 0.01	*U* = 25.00, *p* = 0.063
*Bacteroides_massiliensis*	0.74 ± 0.57	0.01 ± 0.004	*U* = 32.00, *p* = 0.190
*Bacteroides_vulgatus*	0.61 ± 0.23	0	*U* = 25.00, *p* = 0.063
*Roseburia_inulinivorans*	0.55 ± 0.30	0.03 ± 0.03	*U* = 23.00, *p* = 0.043
*Bacteroides_dorei*	0.46 ± 0.22	0.01 ± 0.01	*U* = 25.00, *p* = 0.063

The PICRUSt2 analysis identified six functions related to tryptophan metabolism, specifically TRPSYN‐PWY, PWY‐6629, NADSYN‐PWY, PWY‐5651, PWY‐6505, and PWY‐5655. Notably, PWY‐5655 (L‐tryptophan degradation IX), PWY‐5651 (L‐tryptophan degradation to 2‐amino‐3‐carboxymuconate semialdehyde), and NADSYN‐PWY (NAD biosynthesis II from tryptophan) exhibited significantly higher levels in active snakes (Figure 2B). In addition, a random forest analysis was performed to select the most important bacterial genera based on mean decrease accuracy. This analysis confirmed that *Paeniclostridium*, *Romboutsia*, and *Clostridium_sensu_stricto_1* were the most representative genera for the active snakes, while *Vagococcus* and *Providencia* were the most representative genera for hibernating snakes (Figure 2C).

### 3.2 Analysis of Gut Indoles, Serum Tryptophan Metabolites, and Their Associations With Microbiota

**FIGURE 3:**
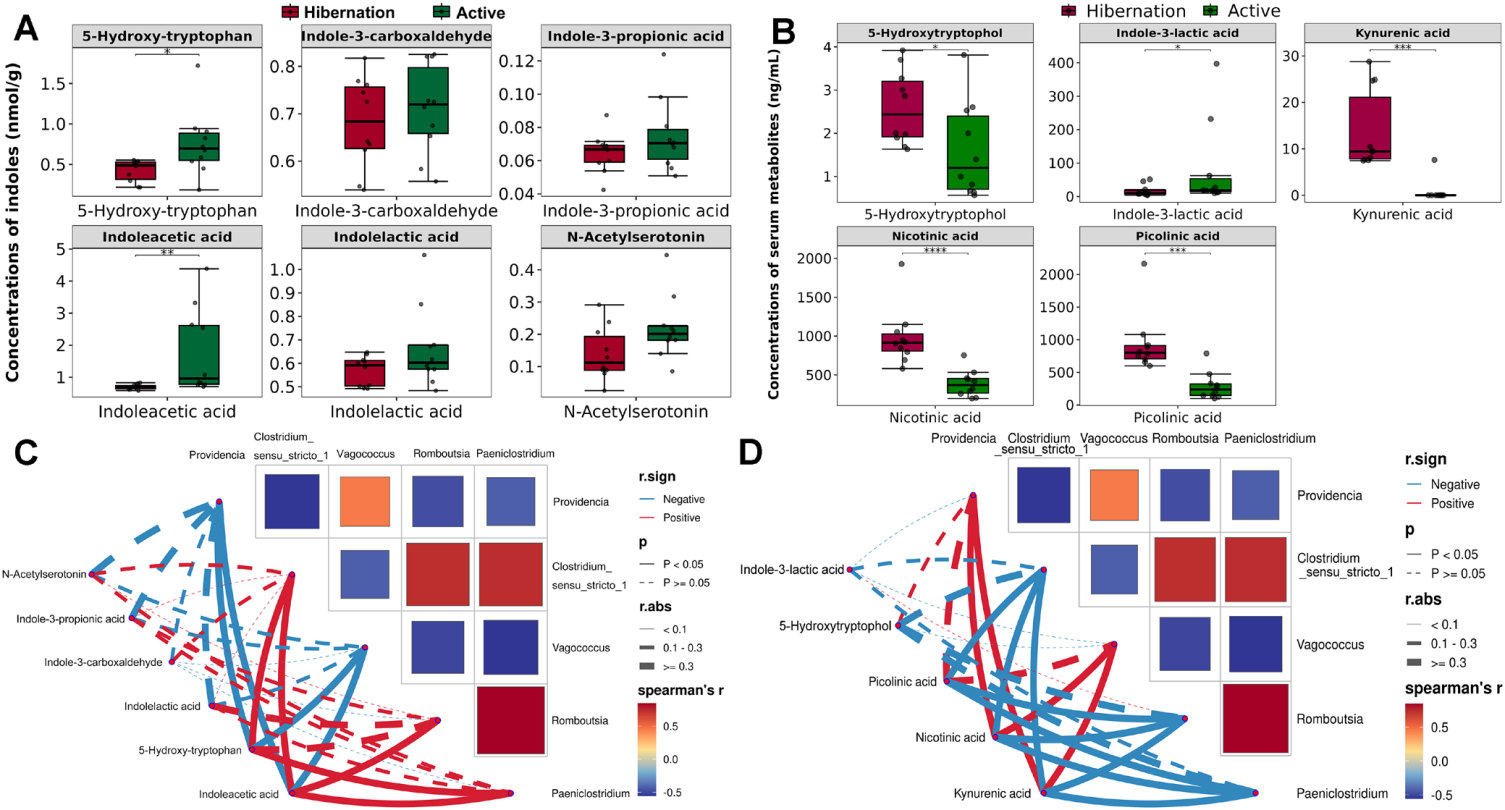
The comparisons of gut indoles (A) and serum tryptophan metabolites (B) with significant differences in Chinese Moccasins between active and hibernation period. *p < 0.05, **p < 0.01, and without “*” means no significant differences. The correlation network heatmap showed the relationship between dominant microbial genera identifeide by Random forest analysis with above gut indoles (C), and serum tryptophan metabolites (D). Blue lines mean negative correlation, and red lines mean positive correlation; dash lines indicate no significant correlation, and solid lines indicate significant correlation.

The targeted metabolome of gut indoles revealed that all six kinds of indoles displayed higher levels in active snakes. Notably, 5‐hydroxy‐tryptophan (Mann–Whitney, *p* = 0.01) and indoleacetic acid (IAA, Mann–Whitney, *p* < 0.01) exhibited significant differences (Figure 3A). The targeted metabolome of serum tryptophan revealed different tryptophan metabolism (Figure S3), while 5 of 24 metabolites exhibited significant differences (Figure S4, *p* < 0.05). Indole‐3‐lactic acid (ILA) displayed significantly higher levels in active snakes (Mann–Whitney, *p* = 0.03), while 5‐hydroxytryptophol (*t*‐test, *p* = 0.04), picolinic acid (PA, *t*‐test, *p* < 0.01), nicotinic acid (NA, *t*‐test, p < 0.01), and KA (Mann–Whitney, *p* < 0.01) were present in higher concentrations in hibernating snakes (Figure 3B). Subsequently, the correlation analysis between representative genera and gut indoles suggested that 5‐hydroxy‐tryptophan was significantly positively correlated with *Paeniclostridium* and *Clostridium_sensu_stricto_1*, and IAA was significantly positively correlated with *Paeniclostridium*, *Romboutsia*, and *Clostridium_sensu_stricto_1*, whereas 5‐hydroxy‐tryptophan and IAA showed significant negative correlation with *Providencia* and *Vagococcus* (Figure 3C). Finally, NA and KA were significantly positively correlated with *Vagococcus* and *Providencia*, while PA, NA, and KA showed significant negative correlation with *Paeniclostridium*, *Romboutsia*, and *Clostridium_sensu_stricto_1* (Figure 3D).

### 3.3 Analysis of Liver Transcriptome Related to Tryptophan Metabolism

**FIGURE 4:**
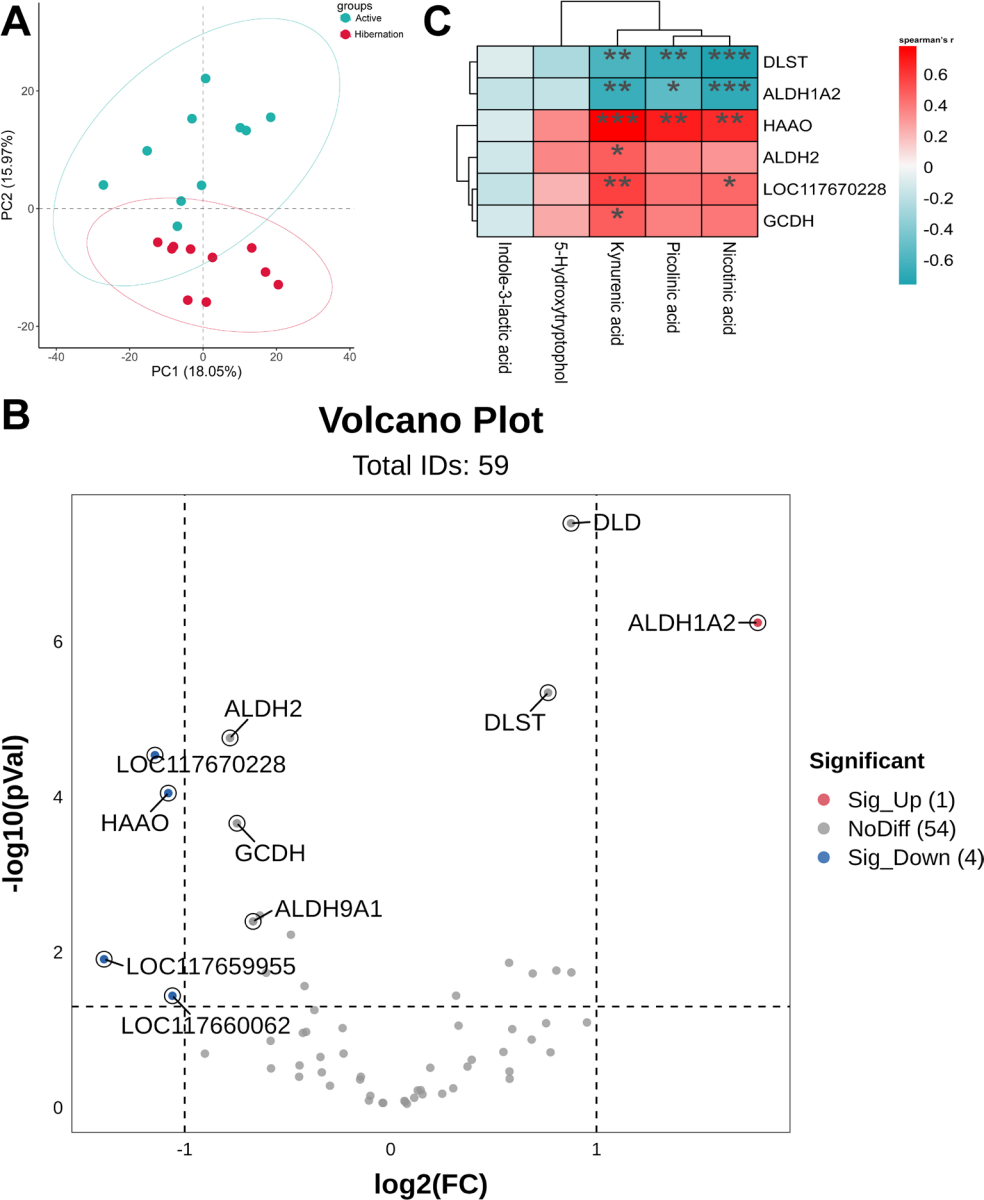
Principal‐component analysis (PCA, A) based on the relative expression of genes related to tryptophan metabolism between active and hibernating Chinese Moccasins. The volcano plots (B) identify the significantly up/down regulated genes in liver of paired comparisons of active versus hibernating snakes. The correlation analysis (C) between differential genes with serum tryptophan metabolites. Blue colors means negative correlation, and red colors means positive correlation. *Means the significant positively or negatively correlation relationships.

After screening the genes associated with tryptophan metabolism (ko00380, ko00403, and ko00901), the principal component analysis plot displayed distinct clusters between two groups (Figure 4A). A total of 59 genes related to tryptophan metabolism were identified, and a volcano plot found some up/downregulated genes. These genes included aldehyde dehydrogenase family 1 member A2 (*ALDH1A2*), LOC117670228 (nicotinamide N‐methyltransferase), and 3‐hydroxyanthranilate 3,4‐dioxygenase (*HAAO*), which showed significant differences; and aldehyde dehydrogenase family 2 (*ALDH2*), dihydrolipoamide dehydrogenase (*DLD*), dihydrolipoamide S‐succinyltransferase (*DLST*), and glutaryl‐CoA dehydrogenase (*GCDH*), which showed *p* < 0.05 but log2 fold change < 1 (Figure 4B). Additionally, correlation analysis between liver genes and serum metabolites revealed that serum PA, NA, and KA were significantly positively correlated with *HAAO*, but negatively correlated with *DLST* and *ALDH1A2* (Figure 4C). Furthermore, KA also showed significantly positive correlation with *ALDH2*, *LOC117670228*, and *GCDH*, while LOC117670228 also has significantly positive correlation only with NA (Figure 4C).

### 3.4 Integrated Analysis Based on Multi‐Omics Data

**FIGURE 5:**
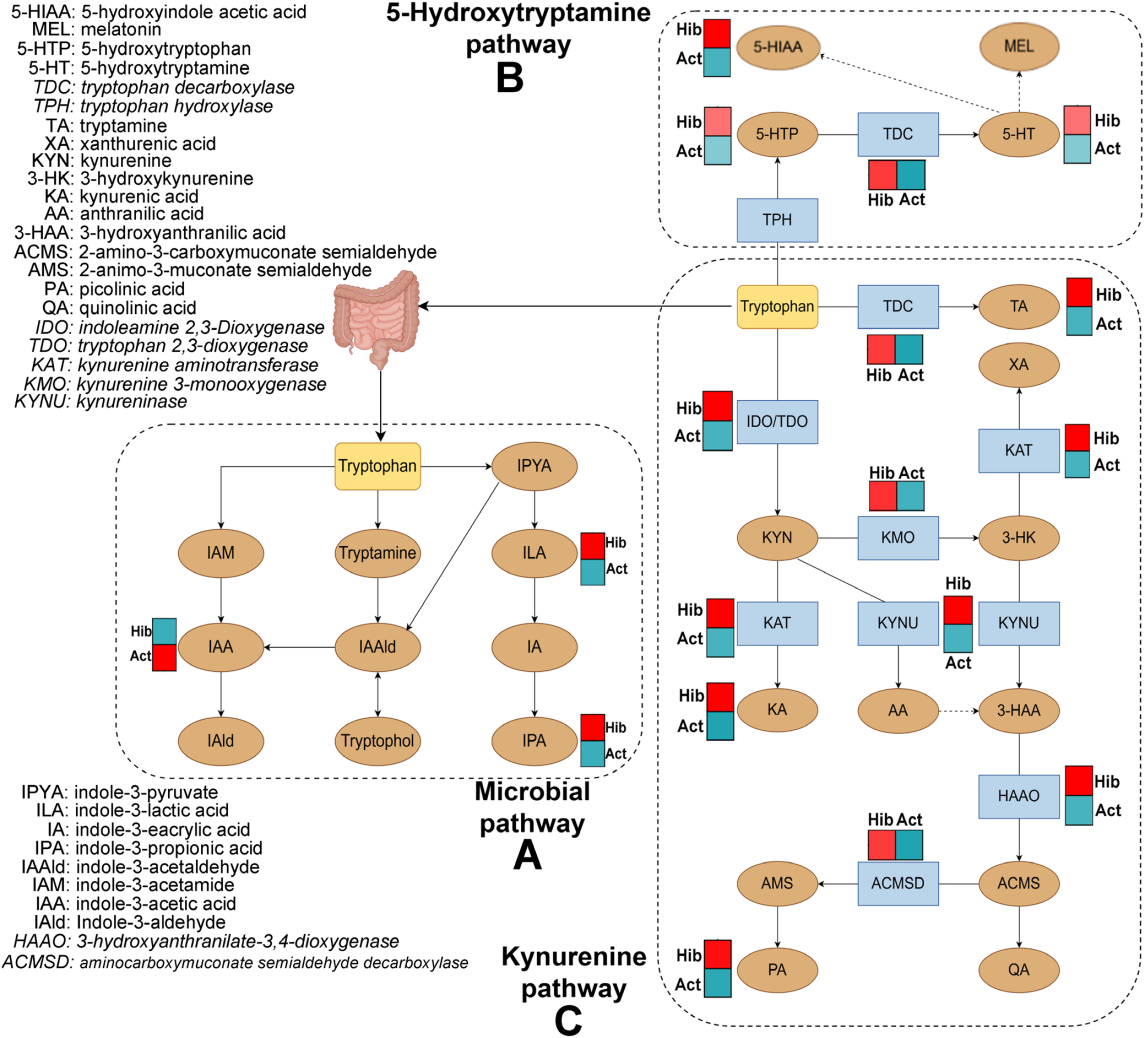
The hand‐painted heatmap pathway maps of three pathways of tryptophan metabolism, including microbial pathway (A), 5‐hydroxytryptamine pathway (B) and kynurenine pathway (C) based on the identified genes, gut indoles and serum tryptophan metabolites. Words in blue boxes are the names of genes, and the words in yellow ellipses are the names of metabolites The heatmaps near them show the differences trends in the gene expression, or metabolites levels/concentrations, and red color means higher levels in hibernating snakes, whereas blue colors means higher levels in active snakes.

Finally, detailed and focused pathway heatmaps for three tryptophan metabolism pathways were constructed: the microbial pathway (Figure 5A), 5‐HT pathway (Figure 5B), and KYN pathway (Figure 5C). As illustrated in Figure 5, most genes and metabolites involved in the 5‐HT and KYN pathways are more abundant in hibernating snakes. Notably, this includes tryptophan decarboxylase (*TDC*), kynurenine 3‐monooxygenase (*KMO*), and *HAAO* genes, as well as products such as 5‐HT, KA, and PA. However, in active snakes, IAA and indole‐3‐propionic acid (IPA) were found at higher levels in intestinal metabolites, while a similar trend was observed for ILA in serum metabolites (Figure 3B). Integrating with the aforementioned correlation analyses (Figure 3C,D), these data collectively suggest that tryptophan metabolism in active snakes is predominantly mediated by the *Paeniclostridium*, *Romboutsia*, and *Clostridium_sensu_stricto_1* for generating IAA and ILA.

## 4 Discussion

### 4.1 Microbial‐Mediated Tryptophan Metabolism Generates Indoles in Active Snakes

This study reveals striking divergence in tryptophan metabolic strategies between hibernating and active snakes, with the latter exhibiting predominant reliance on microbial metabolic pathways. The results indicate a marked restructuring of gut microbiota composition during active phases, characterized by a significant increase in the abundance of Firmicutes and a concurrent decrease in Proteobacteria. At the genus level, 9 of the top 10 abundant microbial taxa exhibited differential enrichment patterns, with *Paeniclostridium*, *Romboutsia*, and *Clostridium_sensu_stricto_1* emerging as keystone taxa in active snakes through Random Forest analysis. These taxa demonstrated strong positive correlations with IAA and ILA levels, and *Clostridium* spp. have been identified as the primary producers of indole (Li et al. 2024), and *Romboutsia* has also been reported by other studies to be positively associated with indole production (Fang et al. 2022), suggesting specialized enzymatic capabilities in tryptophan catabolism (Roager and Licht 2018). Although the literature on tryptophan metabolism in snakes is limited, studies on other animals provide a basis for comparing and interpreting the findings of this study.

Indole derivatives are important end products of tryptophan metabolism and are believed to play crucial roles in modulating various host physiological functions. For instance, IAA and ILA can act as signaling molecules by binding to the aryl hydrocarbon receptor within host cells. This interaction mediates immune cell differentiation, regulates inflammatory responses, and positively influences the barrier function of gastrointestinal epithelial cells (Dong et al. 2020; Powell et al. 2020; Sun et al. 2020). For active snakes—characterized by frequent activity, vigorous predation, and elevated energy metabolism—this microbial‐mediated conversion of metabolites facilitates a mutually beneficial symbiosis between bacteria and host. Additionally, it may reduce the risk of tissue damage induced by high activity by downregulating pro‐inflammatory factors while upregulating anti‐inflammatory ones (Meng et al. 2020). Furthermore, indole compounds might modulate the neuroendocrine axis, affecting the bidirectional communication between the central nervous system and the gut—the so‐called “gut‐brain axis”. This connection can influence behavior, emotion, and adaptive stress responses (Wei et al. 2021; Sun et al. 2022; Zhou et al. 2023). Such signaling mechanisms are vital for processes like feeding, locomotion, and adapting to environmental changes, enabling the host to better manage fluctuations in energy and resource demands. Moreover, research suggests that indole derivatives could regulate the cell cycle, enhance antioxidant defense mechanisms, and maintain gut microbial stability. These functions promote energy metabolism while preventing the colonization of harmful microorganisms and the invasion of pathogens (Andreadou et al. 2002; Negatu et al. 2020).

Overall, in active snakes, the high abundance of Firmicutes and related dominant microbial communities promotes the conversion of tryptophan into IAA and ILA via specific microbial metabolic pathways (Tintelnot et al. 2023). This metabolic mode supplies essential bioactive molecules for regulating local gut immunity and barrier function. Moreover, it offers a novel perspective on the regulation of the host's neuroendocrine and global energy metabolism. Active‐phase snakes experience intense ecological competition and frequent predation, which necessitate a higher level of internal homeostatic regulation compared to hibernating snakes. This microbiota‐mediated metabolic adjustment may represent an evolutionary strategy for coping with high‐activity states.

### 4.2 Host‐Dominated Tryptophan Metabolism via 5‐HT/KYN Pathways Enhances Environmental Resilience in Hibernating Snakes

Conversely, hibernating snakes shift toward host‐controlled tryptophan metabolism, with pronounced activation of 5‐HT and KYN pathways. Transcriptomic profiling identified the upregulation of key enzymes, including *TDC*, *KMO*, and HAAO. These enzymes coordinate to regulate neurotransmitter synthesis (via 5‐HT) and NAD+ biosynthesis (via the KYN pathway), with serum levels of 5‐hydroxyindoleacetic acid (5‐HIAA) and KA exceeding those of active snakes, respectively. Alterations in hepatic expression of enzymes involved in tryptophan metabolism can modulate the circulating availability of this essential precursor, thereby influencing its transport across the blood–brain barrier via competitive amino acid transport systems (Höglund et al. 2019). Studies on hibernating squirrels have shown that hibernation promotes the activation of the brain tryptophan hydroxylase (*TPH*), thereby enhancing the serotonin pathway of tryptophan metabolism (Popova et al. 1993). As an essential neurotransmitter, 5‐HT is involved in central nervous system regulation, mood modulation, and sleep. It also impacts gastrointestinal motility and vascular function (Silber and Schmitt 2010; Gao et al. 2018). Meanwhile, 5‐HTP, an immediate precursor of 5‐HT, is critical for maintaining the dynamic balance of serotonin (Maffei 2020). Given the tissue‐specific metabolic compartmentalization between the liver and brain, such peripheral changes in precursor flux may exert downstream effects on neuronal serotonin synthesis rates without direct transcriptional changes in the central nervous system (Yabut et al. 2019). The level of 5‐HIAA, which is the primary metabolite of 5‐HT, serves as a reflection of the overall activity of serotonin metabolism (De Giovanni et al. 2022). Within the KYN pathway, KA exerts neuroprotective and antiexcitatory effects by suppressing excessive neuronal activation, thereby reducing oxidative stress and inflammatory responses. Additionally, PA demonstrates immunomodulatory and antioxidant properties (Ostapiuk and Urbanska 2022).

The high expression of *TDC*, *KMO*, and *HAAO* indicates that during hibernation, snakes actively regulate the conversion of tryptophan into these neuro‐ and immune‐regulatory bioactive substances. This process provides effective protection in extremely low temperatures, limited energy supply, and slow metabolism (Dang et al. 2023). Notably, the regulatory actions of 5‐HT and its metabolites help maintain stable neurotransmission, preventing neuronal dysfunction under low‐temperature conditions (Okaty et al. 2019). Moreover, the roles of KA and PA in promoting anti‐inflammatory and antioxidant effects provide a molecular shield against cellular damage due to prolonged exposure to low temperatures or reduced metabolic rates (Sheipouri et al. 2012).

Importantly, this metabolic remodeling is a unidirectional mechanism for energy conservation. Besides, it represents a multifaceted, system‐wide regulatory process that integrates coordinated functions among the nervous, endocrine, and immune systems. By integrating these functions, hibernating snakes enhance their ability to withstand internal environmental challenges, thereby maintaining physiological homeostasis even under prolonged adverse external conditions. Considering that hibernation is often associated with rapid temperature declines and food scarcity, this metabolic adaptation strategy—relying on the 5‐HT and KYN pathways—helps maintain a constant supply of bioactive molecules. It may also enhance the host's antioxidant, anti‐inflammatory, and neuroprotective capabilities by activating protective cellular signals and regulating gene expression (Zhen et al. 2022).

## 5 Conclusion

In conclusion, these findings collectively demonstrate how seasonal metabolic plasticity in tryptophan catabolism serves as a crucial hub for coordinating energy homeostasis, oxidative defense, and neural adaptation in snakes. Furthermore, identifying the tryptophan‐catabolizing, indole‐producing microbes provides essential data for developing probiotic‐based interventions that could enhance the success rates of snake overwintering. Investigating the mechanisms involved in this process enhances our understanding of ectotherm survival adaptations, providing a novel perspective into the evolutionary optimization of metabolic strategies across vertebrate taxa. However, while many interpretations draw primarily on evidence from mammals and other model organisms, they should be considered as tentative and subject to confirmation through future experimental validation in reptiles.

## Author Contributions

**Yuting Wei:** data curation (lead), formal analysis (lead), investigation (lead), methodology (lead), software (lead), validation (equal), visualization (lead), writing – original draft (lead). **Zexiu Zhang:** conceptualization (equal), resources (equal). **Xiaohong Lin:** conceptualization (equal), writing – review and editing (equal). **Gangning Wei:** formal analysis (equal), methodology (equal), validation (equal), visualization (equal), writing – original draft (equal). **Pengyue Zhang:** conceptualization (equal), project administration (equal), writing – review and editing (equal). **Huirong Mao:** conceptualization (equal), validation (equal), writing – review and editing (equal). **Biao Chen:** conceptualization (equal), validation (equal), writing – review and editing (equal). **Jianhao Ji:** conceptualization (equal), methodology (equal), writing – review and editing (equal). **Yunlin Zheng:** project administration (equal), writing – review and editing (equal). **Zhiyi Luo:** conceptualization (equal), project administration (equal), writing – review and editing (equal). **Xiaolong Hu:** conceptualization (lead), funding acquisition (lead), project administration (lead), resources (lead), supervision (lead), validation (lead), writing – review and editing (lead).

## Conflicts of Interest

The authors declare no conflicts of interest.

## Supporting information


**Figure S1:** The comparison of microbial α‐diversity between hibernation and active snakes.
**Figure S2:** The core microbe analysis of samples from active and hibernating snakes. Flower figures show the unique and shared numbers (Core) of amplicon sequence variants (ASVs) among active snakes (A, AM1‐AM10) and hibernating snakes (C, HM1‐HM10). Pie plots exhibit the composition of core microbial community at five levels for active snakes (B) and hibernating snakes (D).
**Figure S3:** Orthogonal projections to latent structures discriminant analysis (OPLS‐DA) of serum tryptophan metabolites between hibernation and active snakes.
**Figure S4:** The comparison of serum tryptophan metabolites with significant differences between hibernation (green) and active (red) snakes.
**Table S1:** The initial body weight and age of snakes in hibernation and active groups.
**Table S2:** The α‐diversity values of gut microbiota in hibernation (H1‐H10) and active snakes (A1‐A10).

## Data Availability

The raw sequence data of transcriptome (CRA025485) and microbiome (CRA025484) reported in this paper have been deposited in the Genome Sequence Archive in National Genomics Data Center, China National Center for Bioinformation/Beijing Institute of Genomics, Chinese Academy of Sciences and that are publicly accessible at https://ngdc.cncb.ac.cn/gsa.
