# Supplementary material for: Seasonal Plasticity in Tryptophan Metabolism Provides New Insights Into Physiological Adaptation in Snake Hibernation
Source: Ecol Evol. 2025 Sep 17;15(9):e72202. doi: 10.1002/ece3.72202 (PMC12443614; doi:10.1002/ece3.72202)
Supplement: Supplementary file 1 — Figure S1: The comparison of microbial α‐diversity between hibernation and active snakes. Figure S2: The core microbe analysis of samples from active and hibernating snakes. Flower figures show the unique and shared numbers (Core) of amplicon sequence variants (ASVs) among active snakes (A, AM1‐AM10) and hibernating snakes (C, HM1‐HM10). Pie plots exhibit the composition of core microbial community at five levels for active snakes (B) and hibernating snakes (D). Figure S3: Orthogonal projections to latent structures discriminant analysis (OPLS‐DA) of serum tryptophan metabolites between hibernation and active snakes. Figure S4: The comparison of serum tryptophan metabolites with significant differences between hibernation (green) and active (red) snakes. Table S1: The initial body weight and age of snakes in hibernation and active groups. Table S2: The α‐diversity values of gut microbiota in hibernation (H1‐H10) and active snakes (A1‐A10). [file ECE3-15-e72202-s001.docx]

**Figure S1** The comparison of microbial α-diversity between hibernation and active snakes.


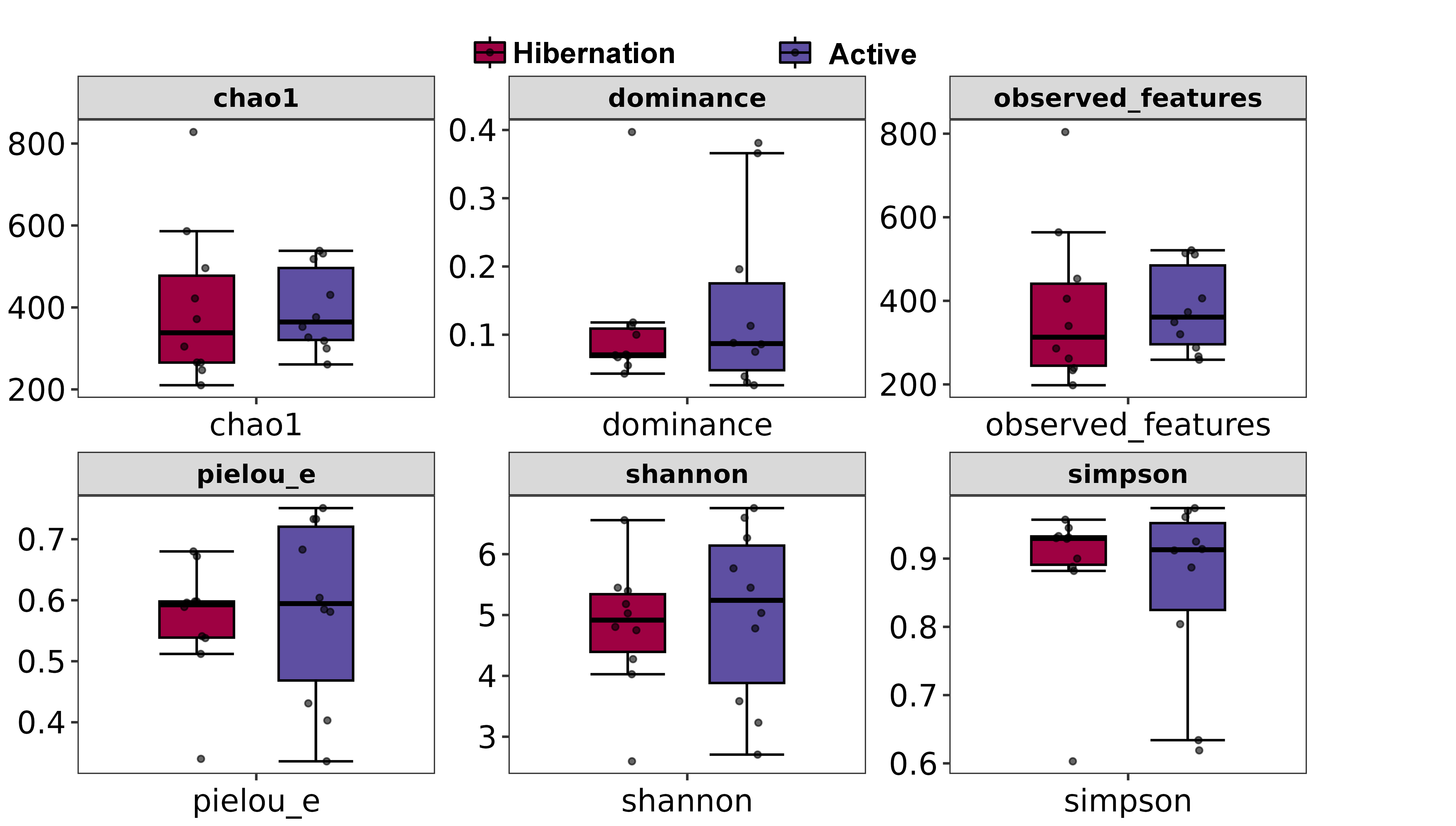


**Figure S2** The core microbe analysis of samples from active and hibernating snakes. Flower figures show the unique and shared numbers (Core) of amplicon sequence variants (ASVs) among active snakes (A, AM1-AM10) and hibernating snakes (C, HM1-HM10). Pie plots exhibit the composition of core microbial community at five levels for active snakes (B) and hibernating snakes (D).


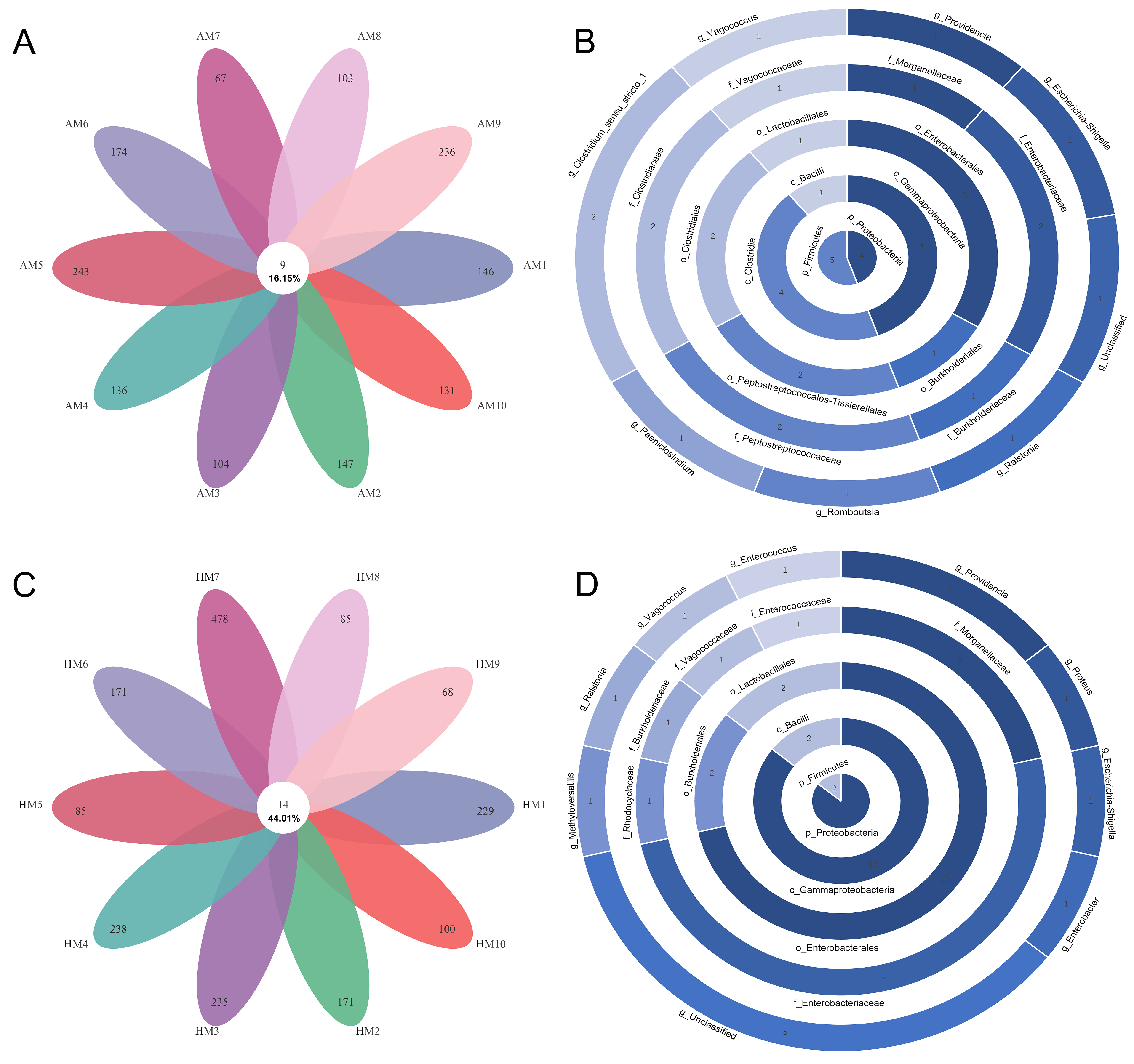


**Figure S3** Orthogonal projections to latent structures discriminant analysis (OPLS-DA) of serum tryptophan metabolites between hibernation and active snakes.


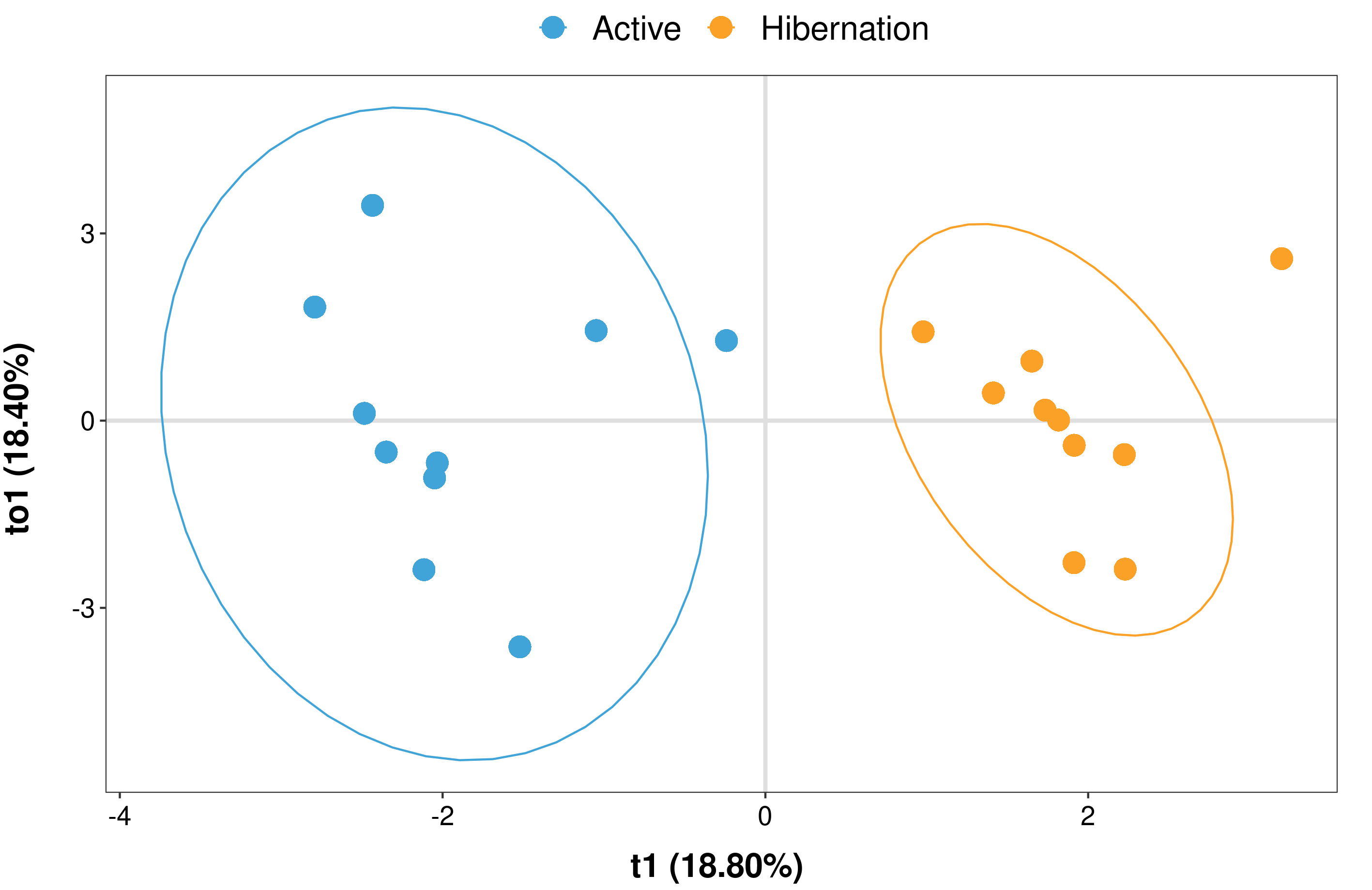


**Figure S4** The comparison of serum tryptophan metabolites with significant differences between hibernation (green) and active (red) snakes.


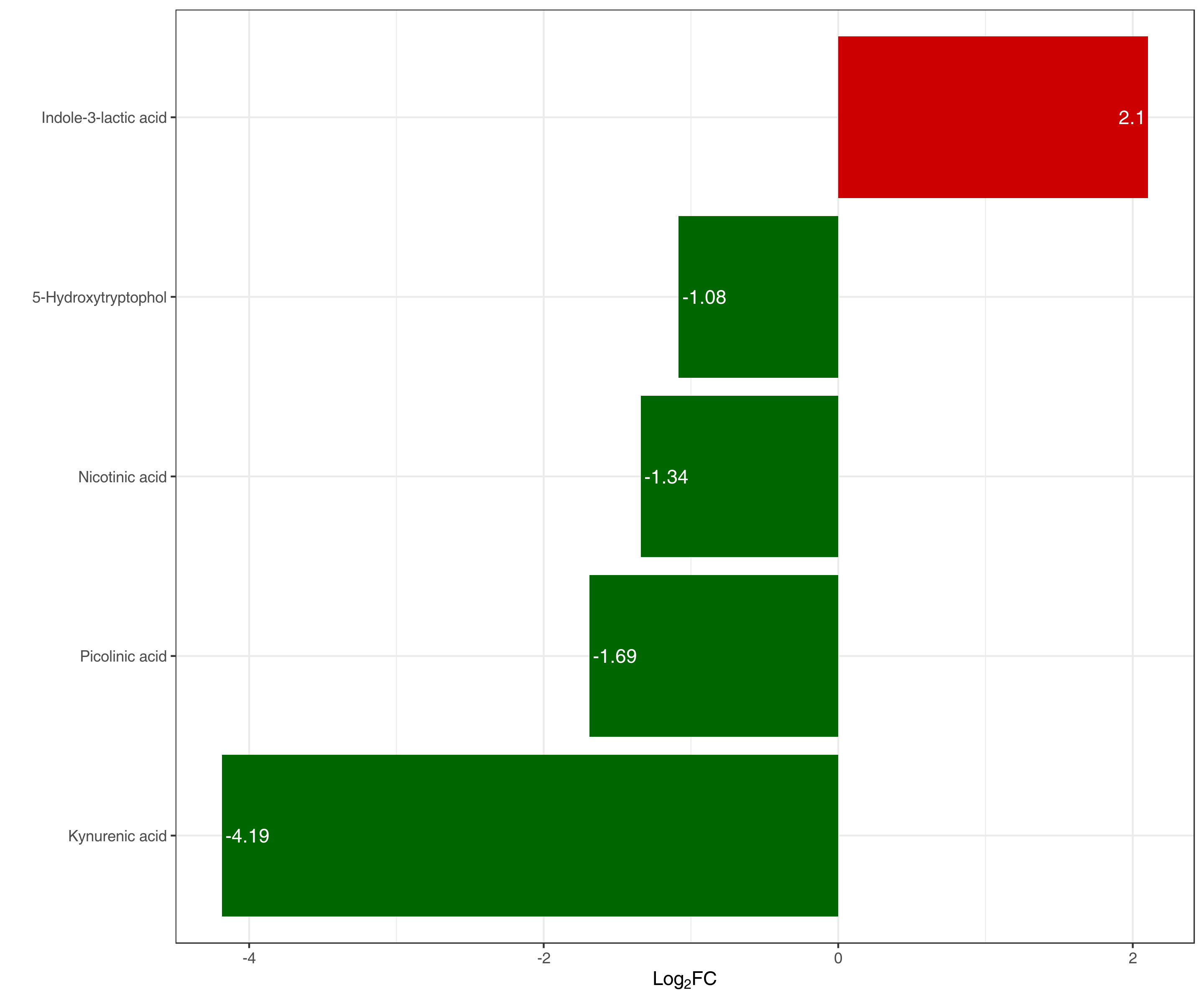


**Table S1** The initial body weight and age of snakes in hibernation and active groups.

| Groups | Age (years old) | Bady weight (kg) |
| --- | --- | --- |
| Active group | 1.5 | 0.94 |
|  | 2 | 1.11 |
|  | 1.5 | 0.95 |
|  | 1.5 | 0.89 |
|  | 1.5 | 0.90 |
|  | 1.5 | 0.87 |
|  | 2 | 1.05 |
|  | 1.5 | 0.86 |
|  | 1.5 | 0.94 |
|  | 2 | 0.99 |
| Hibernation group | 2 | 1.13 |
|  | 1.5 | 0.83 |
|  | 1.5 | 0.95 |
|  | 1.5 | 0.96 |
|  | 1.5 | 0.86 |
|  | 2 | 0.98 |
|  | 1.5 | 0.87 |
|  | 1.5 | 0.91 |
|  | 1.5 | 0.89 |
|  | 2 | 0.81 |

**Table S2** The α-diversity values of gut microbiota in hibernation (H1-H10) and active snakes (A1-A10).

| Groups | Snakes ID | Chao1 | dominance | observed_features | pielou_e | Shannon | Simpson |
| --- | --- | --- | --- | --- | --- | --- | --- |
| Active group | A1 | 422 | 0.071 | 405 | 0.598 | 5.181 | 0.929 |
|  | A2 | 372 | 0.069 | 340 | 0.598 | 5.028 | 0.931 |
|  | A3 | 496 | 0.100 | 453 | 0.538 | 4.750 | 0.900 |
|  | A4 | 586 | 0.067 | 564 | 0.596 | 5.449 | 0.933 |
|  | A5 | 247 | 0.118 | 239 | 0.541 | 4.275 | 0.882 |
|  | A6 | 266 | 0.055 | 262 | 0.672 | 5.396 | 0.945 |
|  | A7 | 828 | 0.043 | 804 | 0.680 | 6.559 | 0.957 |
|  | A8 | 265 | 0.112 | 234 | 0.512 | 4.027 | 0.888 |
|  | A9 | 210 | 0.397 | 198 | 0.340 | 2.596 | 0.603 |
|  | A10 | 305 | 0.070 | 286 | 0.589 | 4.805 | 0.930 |
| Hibernation group | H1 | 319 | 0.075 | 288 | 0.585 | 4.780 | 0.925 |
|  | H2 | 353 | 0.088 | 349 | 0.683 | 5.767 | 0.912 |
|  | H3 | 300 | 0.366 | 267 | 0.336 | 2.706 | 0.634 |
|  | H4 | 538 | 0.113 | 521 | 0.604 | 5.449 | 0.887 |
|  | H5 | 431 | 0.086 | 406 | 0.581 | 5.034 | 0.914 |
|  | H6 | 327 | 0.196 | 320 | 0.431 | 3.584 | 0.804 |
|  | H7 | 261 | 0.381 | 259 | 0.403 | 3.231 | 0.619 |
|  | H8 | 376 | 0.030 | 373 | 0.733 | 6.266 | 0.970 |
|  | H9 | 518 | 0.039 | 514 | 0.733 | 6.598 | 0.961 |
|  | H10 | 532 | 0.026 | 511 | 0.751 | 6.757 | 0.974 |
